# Evidence for the beneficial effect of perceptual grouping on visual working memory: an empirical study on illusory contour and a meta-analytic study

**DOI:** 10.1038/s41598-018-32039-4

**Published:** 2018-09-14

**Authors:** Jiaofeng Li, Jiehui Qian, Fan Liang

**Affiliations:** Sun Yat-Sen University, Department of Psychology, Guangzhou, 510000 China

## Abstract

The capacity of visual working memory (VWM) is found to be extremely limited. Past research shows that VWM can be facilitated by Gestalt principles of grouping, however, it remains controversial whether factors like the type of Gestalt principles, the characteristics of stimuli and the nature of experimental design could affect the beneficial effect of grouping. In particular, studies have shown that perceptual grouping could improve memory performance for a feature that is relevant for grouping, but it is unclear whether the same improvement exists for a feature that is irrelevant for grouping. In this article, an empirical study and a meta-analytic study were conducted to investigate the effect of perceptual grouping on VWM. In the empirical study, we examined the grouping effect by employing a Kanizsa illusion in which memory items were grouped by illusory contour. We found that the memory performance was improved for the grouped items even though the tested feature was grouping irrelevant, and the improvement was not significantly different from the effect of grouping by physical connectedness or by solid occlusion. In the meta-analytic study, we systematically and quantitatively examined the effect of perceptual grouping on VWM by pulling the results from all eligible studies, and found that the beneficial grouping effect was robust but the magnitude of the effect can be affected by several moderators. Factors like the types of grouping methods, the duration and the layout of the memory display, and the characteristics of the tested feature moderated the grouping effect, whereas whether employing a cue or a verbal suppression task did not. Our study suggests that the underlying mechanism of the grouping benefit may be distinct with regard to grouping relevancy of the to-be-stored feature. The grouping effect on VWM may be independent of attention for a grouping relevant feature, but may rely on attentional prioritization for a grouping irrelevant feature.

## Introduction

One of the major research topics in cognitive psychology is how information is temporarily retained and stored in working memory. In the recent decades, working memory related to processing visual information has gained a growing interest and studies on visual working memory (VWM) are extensive^1–7^. It has been suggested that VWM is closely related to fluid intelligence^8,9^, and helps to maintain perceptual stability across variations in retinal images as a result of eye, head or body movements, thus is crucial for visual perception and cognition.

Previous research showed that visual working memory is severely limited in capacity, with only 3–4 items able to be stored from a given display^1,4,10–13^. As for the unit of storage in VWM, it is commonly accepted to be object rather than feature. For example, some research showed that objects defined by a conjunction of multiple features could be retained in VWM as well as single-feature objects^4,12^, a theory known as the fixed-resolution ‘slot’ model. However, other studies suggested that the number of items stored in VWM and the precision of the stored representations might vary substantially across different kinds of stimuli^1,3^.

Several cognitive factors may contribute in overcoming the capacity limitation of VWM. For example, evidence showed that VWM could be enhanced with the aid of long term memory^14^, and factors like familiarity^15^, attentional selection^16,17^ and perceptual grouping^5,18^ also seemed to affect VWM. In particular, perceptual grouping, which was established by the pioneering work of the Gestalt psychologists^19^, demonstrates several simple grouping rules that may influence our perception, such as proximity, similarity, good continuation, connectedness, etc. For example, *proximity* refers to grouping objects according to spatial nearness; and *similarity* refers to grouping based on repetition of similar features like color or shape. These Gestalt principles make the grouped objects appear to ‘belong together’ and be processed as a whole, and therefore facilitate visual perception.

Among these grouping principles, proximity, similarity, and connectedness were most frequently tested with various perceptual tasks. Despite the general improvement in performance, research showed that these principles might vary in grouping effectiveness. Han *et al*.^20^ found that discrimination of stimuli grouped by proximity was faster than that grouped by shape similarity, and the error rates were higher for shape similarity grouping^20^. However, Ben-Av & Sagi^21^ found that although proximity grouping was perceived much faster than similarity grouping, similarity gradually overtook proximity with increasing processing time, and became to dominate grouping^21^. Another finding showed that proximity and similarity might have additive benefit on performance^22^. Quinlan & Wilton^23^ discriminated the effects of similarity grouping by testing two features, color and shape, and found that proximity and color similarity grouping had persuasive beneficial effect on the performance of a grouping discrimination task, while only weak evidence was found for shape similarity grouping^23^. In addition, although there are some controversies^21,24,25^, perceptual grouping seems to occur at a preattentive stage of visual processing^26–30^. Studies suggested that the performance in perceptual discriminations remained accurate in the absence of attention^31–33^. Therefore, perceptual grouping appears to happen at an early stage in the processing stream independent of attentional resources.

There is evidence confirming that perceptual grouping benefits visual working memory as well. Woodman *et al*.^18^ found that proximity and connectedness improved VWM by using a precuing change detection task^18^. In their study, the briefly presented memory array was preceded by a peripheral cue which could direct attention to one of the memory items. After a retention interval with a blank screen, a probe was shown and the participants were instructed to judge whether there was a change between the probe and the tested memory item. The logic was when one element of the group was stored in VWM (the cued item), other elements of the same group were likely to be stored as well. In other words, items grouped together tended to be stored together. In addition, studies showed that several other grouping principles benefited VWM performance as well^5,34–37^. For example, Peterson & Berryhill^5^ found that color similarity facilitated VWM but only when the similar stimuli were proximal^5^. Gao *et al*.^38^ tested the grouping effect of collinearity on VWM using a sequential display paradigm^38^. The memory items in their study were solid disks that contained a rectangular gap, and could be grouped into chunks with a percept of a virtual elongated rectangle occluding two blacks disks. The authors found that the performance for memorizing the orientation of memory items was better when the disks were grouped. Other research also showed that the spatial configuration can influence VWM storage^39–45^. However, despite the converging evidence for the beneficial effect of perceptual grouping, previous findings are inconsistent regarding the degree to which the memory performance can be enhanced by a particular grouping principle, and there is no consensus on whether multiple grouping principles could jointly influence VWM or work independently with each other. Moreover, it is unclear whether the grouping effect on VWM can be influenced by experimental settings, such as tasks and stimuli used in a study. Therefore, it is beneficial to conduct a quantitative and comprehensive review for the relevant studies upon this topic.

This article included one empirical study and a meta-analytic study. In the empirical study, we investigated the grouping effect of illusory contour on VWM. Although Gao’s study demonstrated improved memory performance for the orientation of the gapped disks that formed an illusory rectangle^38^, note that the percept of the illusory rectangle relied critically on the perception of the disk’s orientation, i.e., the tested feature – orientation – was directly related to the formation of the illusory figure and thus was grouping relevant. It is unclear whether this beneficial grouping effect could be generalized to other feature irrelevant for the formation of the illusory figure and thus grouping. In the present study, the to-be-remembered items were colored circular sectors that formed a Kanizsa illusion and thus were perceived as having an illusory figure occluding the disks. We investigated the effect of perceptual grouping by illusory contour on VWM by testing the color of the sectors, and we predicted that the items grouped by illusory contour would be better remembered, regardless of the relevancy of the tested feature for forming an Kanizsa illusion. In the meta-analysis, we aimed to systematically examine the effect of perceptual grouping on VWM by pulling the results from all eligible studies. In particular, we combined and summarized the effects of different grouping principles reported to date, and then compared the effects by several potential moderators. Moderator analyses might have important indications on the underlying mechanisms involved in the process of perceptual grouping and serve to direct and focus the future research in the related topics.

## Empirical Study

Previous study showed that the memory performance for the orientation of the disks could be enhanced in a Kanizsa illusion^38^. However, it is possible that grouping by illusory contour only facilitates the memory for a grouping relevant feature. Whether the grouping effect varies with the relevancy of the tested feature and grouping remains uncertain. In this study, three experiments were carried out to investigate the grouping effect of closure, which was induced by an illusory contour, on memorizing a grouping irrelevant feature. In addition, the magnitude of the grouping effect of closure was compared with that of connectedness to examine their differences in grouping effectiveness on VWM.

### Method

#### Participants

Sixty-six students (mean age = 20.1 ± 2.4) from Sun Yat-Sen University (SYSU) with normal or corrected-to-normal vision took part in Experiment 1 for pay; fifteen students (mean age = 21.2 ± 1.3) took part in Experiment 2; and another fifteen (mean age = 20.8 ± 1.6) took part in Experiment 3. All of them were naive to the purpose of the study. This research was approved by Sun Yat-Sen University Institutional Review Board (IRB). The study was carried out in accordance with the relevant guidelines and regulations. Written informed consent approved by the IRB was obtained from each participant prior to all the experiments.

#### Apparatus

The stimuli were viewed against a uniform gray background (35.4 *cd*/*m*^2^) on a 23-inch HP proDisplay P231 monitor. The display resolution was set to 1920 × 1080 pixels, with a refresh rate of 60 Hz. For the typical viewing distance of 70 cm, a pixel subtended approximately 1 arcmin.

#### Stimuli and Procedures in Experiment 1

**Figure 1:**
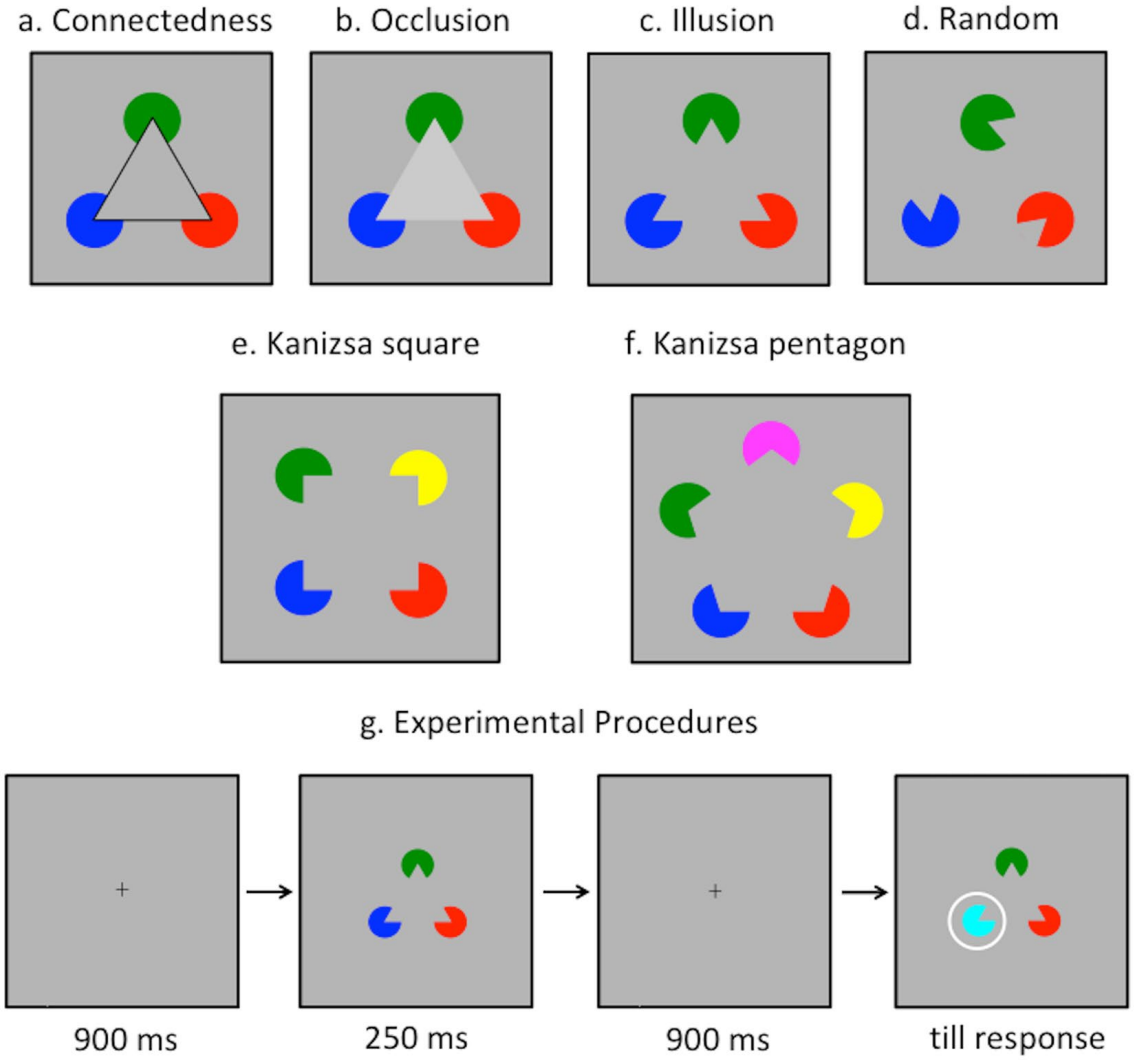
Stimuli and Procedures in Experiment 1. The upper panels show the exemplar stimuli with a set size 3 in the connectedness (**a**), occlusion (**b**), illusion (**c**), and random orientation (**d**) conditions. The middle panels show the exemplar stimuli in the illusion condition with a set size of 4 (**e**) and 5 (**f**). Task sequences are shown in (**g**).

The memory display was composed of a set of colored circular sectors (Fig. 1a–f). The color of each item was selected at random from seven colors: red, green, blue, yellow, magenta, cyan, black. Its size subtended approximately 1.2° of visual angle. The distance between the centers of any two neighbouring items was 2.4°. Three set sizes were used in the experiment. When the set size was 3, the items constituted a Kanizsa triangle and thus were perceived as a large triangle occluding the three disks (Fig. 1c); when the set size was 4, the items constituted a Kanizsa square (Fig. 1e); when the set size was 5, the items constituted a Kanizsa pentagon (Fig. 1f). Four configuration conditions were examined: in the connetedness condition, the illusory figure was outlined so that the circular sectors were physically connected by the black lines (Fig. 1a); in the occlusion condition, the color of the illusory figure was brighter (102.2 *cd*/*m*^2^) than the bakcground (Fig. 1b); in the illusion condition, there was no enhancement to the illusory figure (Fig. 1c); in the random orientation condition, the orientation of each circular sector was selected at random from 0° to 360° so that no illusory Kanizsa figure was perceived. The display was presented at the center of the screen. During the test phase, the whole display were presented again with one probe, marked by a white circle, either changing its color to the remaining color that had not been used in the memory display, or being kept the same.

Observers were seated in a dark room to complete the whole experiments. They were trained for a short time (2–5 min) to get acquainted with the stimuli and the task. Each trial began with a 900 ms presentation of a fixation cross that subtended 0.3° × 0.3° at the center of the screen (Fig. 1g). The memory array was then presented for 250 ms. It was followed by a 900 ms blank retention interval and then the test phase. The test display remained on the screen until observers responded. After the response, a 1,000 ms blank intertrial interval was presented before the next trial.

Sixty-six observers were randomly assigned to the different set-size conditions. For each set size, twenty-two observers participated in all four configuration conditions with the order counterbalanced among the observers. The observers were informed that the marked memory items might change its color and their task was to judge whether there had been a change. If ‘a change’ was perceived, they pressed the right arrow on the keyboard; otherwise, the left arrow. They were encouraged to remember all the items as well as possible. On 50% of the trials, the color of the probe changed. Each observer received 120 trials for each condition, yielding a total of 480 trials. The order of the ‘change’ and ‘no change’ trials in each condition were randomized during the experiment.

#### Stimuli and Procedures in Experiment 2

**Figure 2:**
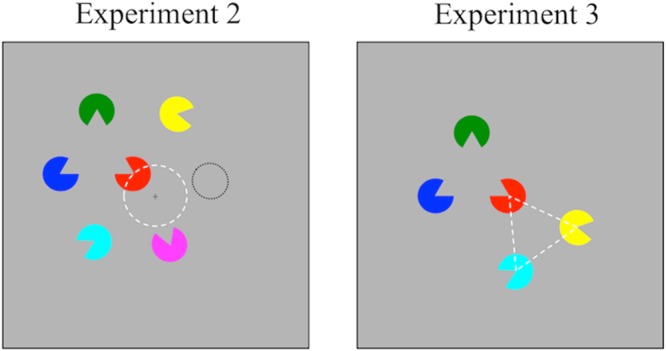
Stimuli in Experiment 2 & 3. Left panel: Experiment 2, the illusion condition. The item positioned at the center of the whole memory display jiggered along a radius of 1°, indicated by a white dashed circle, from the center of the screen across trials. The black dotted circle indicates another possible location for the ungrouped item in this trial. Right panel: Experiment 3. Both the grouped items and the ungrouped item formed a configuration of triangle with the central item, but their orientations in relation to the central item were different. The lines and circles were for demonstration and not shown in the formal experiments.

The experimental procedure was identical to Experiment 1, except the following changes. The memory display was composed of six colored circular sectors, with three of the items constituting a Kanizsa triangle (Fig. 2, the left panel). The orientation of the other three sectors was randomly selected. The distance between the centers of any two nearest neighbouring items was 2.4°. An item was positioned at the center of all memory items and jiggered along a radius of 1° from the center of the screen across trials. The entire display was presented within a radius of 5°. The test item was one of the three items that formed the Kanizsa triangle in half of the trials (the grouped condition), and was one of the items outside the triangle in the other half (the ungrouped condition). The connectedness, occlusion, and illusion conditions were tested. Each observer received 180 trials for each condition, yielding a total of 540 trials.

#### Stimuli and Procedures in Experiment 3

The experimental procedure was identical to the illusion condition in Experiment 2, except the following changes. The memory display was composed of five colored circular sectors, with one placed at the center of the screen (Fig. 2, the right panel). Two of the peripheral sectors (grouped items) and the central sector constituted a Kanizsa triangle; the other two peripheral sectors (ungrouped items) and the central sector also located at the vertices of a similar triangle, but their orientations were randomly selected. The distance between the centers of any grouped and ungrouped items was greater than 2.4°, in order to avoid proximity grouping between the grouped and ungrouped items. The only difference between the grouped and ungrouped items was their orientations in relation to the central item. The test item was the central item in one third of the trials, one of the grouped items in one third of the trials, and one of the ungrouped items in the rest of the trials. Each observer received a total of 180 trials.

### Results

#### Experiment 1

Three grouping configurations were examined to reveal the possible difference in the grouping effect between an illusory figure and a realistic one. In the connectedness condition, the contours of the illusory occluder were outlined so the memory items were physically connected by the black lines. This condition aimed to replicate the grouping effect of connectedness on VWM^18^ and served as a ‘grouped’ comparison or baseline for the other configuration conditions. In the occlusion condition, the color of the Kanizsa figure was manipulated to be brighter than the background. This was because of an often-noted percept that the illusory figure is perceived to be slightly brighter than the background. The percept may result from the mechanism of lightness constancy: the brain infers that the occluder, though has the same shade of gray as the background, must be brighter because it is closer to observer and thus reflects more lights. In this condition, we attempted to test whether the grouping effect varied with the solidness of the occluder. No enhancement was applied to the illusory figure in the illusion condition. In the random orientation condition, each sector had a random orientation thus did not form a Kanizsa illusion.

**Figure 3:**
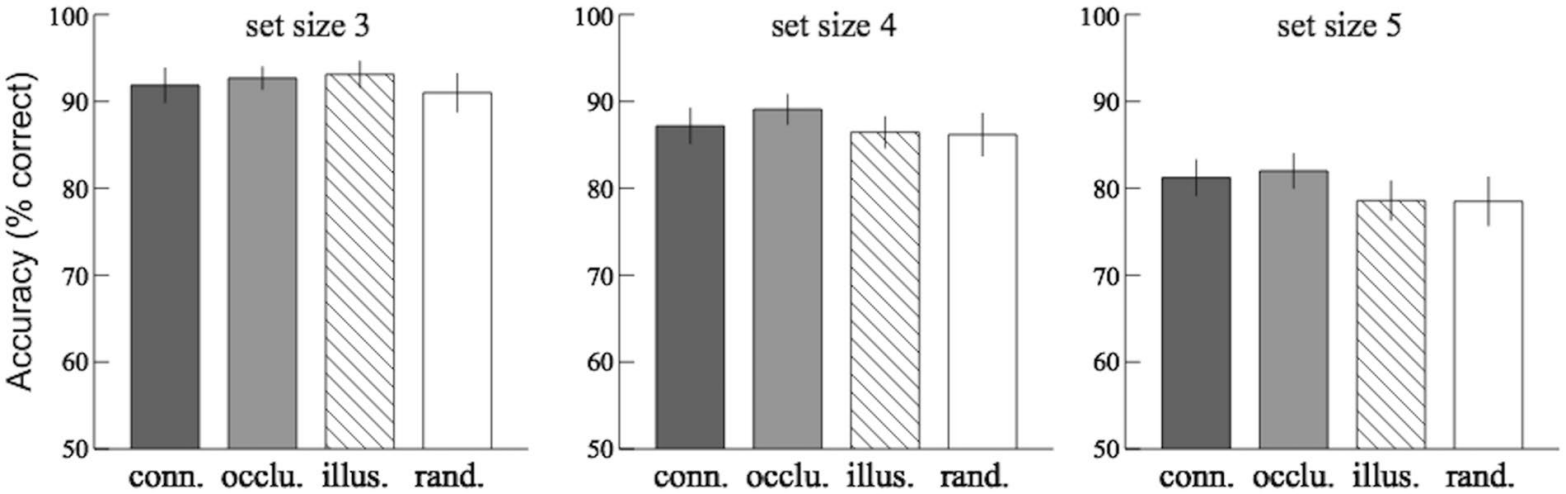
Results of Experiment 1: comparison between the average change detection accuracies for the four configuration conditions – connectedness (conn.), occlusion (occlu.), illusion (illus.), and random orientation (rand.). Error bars indicated standard errors of the mean.

The results are shown in Fig. 3. A 4 × 3 (configuration × set size) mixed-design analysis of variance (ANOVA) was conducted. There was a significant difference among the change detection accuracy for the three set sizes [$F(2,63)=15.02,p < 0.001,{\eta }_{p}^{2}=0.32$]. Post-hoc comparison showed that there were significant differences in accuracy between set size 3 and 5, [*F*(1, 42) = 32.07, *p* < 0.001, Bonferroni corrected, ${\eta }_{p}^{2}=0.43$], and set size 4 and 5, [*F*(1, 42) = 8.66, *p* = 0.006, Bonferroni corrected, ${\eta }_{p}^{2}=0.17$]. There was no significant difference between the results for the four configuration conditions, [$F(3,189)=2.14,p=0.09,{\eta }_{p}^{2}=0.03$]. No interaction was found, [$F(6,189)=0.52,p=0.79,{\eta }_{p}^{2}=0.02$].

In addition, since we expected the memory performance for the three grouping configuration conditions to be better than that for the random orientation condition, planned contrast comparisons were performed for each grouping configuration condition vs. the random orientation condition. Consistent with the results of ANOVA, there was no significant difference in the accuracy for connectedness condition vs. random orientation condition, [$$F(1,63)=1.52,p=0.22,{\eta }_{p}^{2}=0.02$$]; occlusion condition vs. random orientation condition, [$$F(1,63)=3.90,$$$$p=0.063,{\eta }_{p}^{2}=0.05$$]; or illusion condition vs. random orientation condition, [$$F(1,63)=0.38,$$ $$p=0.54,{\eta }_{p}^{2}$$ $$=\,0.006$$].

#### Experiment 2

Experiment 1 showed that the change detection accuracy was not significantly improved in the three grouping configuration conditions, even when the sectors were explicitly connected by lines in the connectedness condition. It was possible that since each sector was positioned at a vertex of the Kanizsa figure in all four conditions, the general configuration of the display dominated the perception after extensive repetitions of trials. In other words, observers might overwhelmingly perceive the whole figure and ignore the detailed differences such as whether the sectors were physically connected or not. Another possible explanation was that the grouping effect on VWM might involve a process of competing for memory resources between grouped and ungrouped items. In other words, perceptual grouping might induce a bias toward grouped item so that it could receive more resources and be better stored than ungrouped items. Since the grouped and ungrouped items were tested in separate conditions in Experiment 1, the competition process was not triggered and the grouping effect was not observed. Therefore, in Experiment 2, the grouped and the ungrouped items were tested in a single trial to induce potential competition for resources and the ungrouped items were located at pseudorandom locations to eliminate the perception of a whole figure. By this manipulation, we predicted that if the grouped items received more memory resource, the performance for the grouped items would be better than for the ungrouped items.

**Figure 4:**
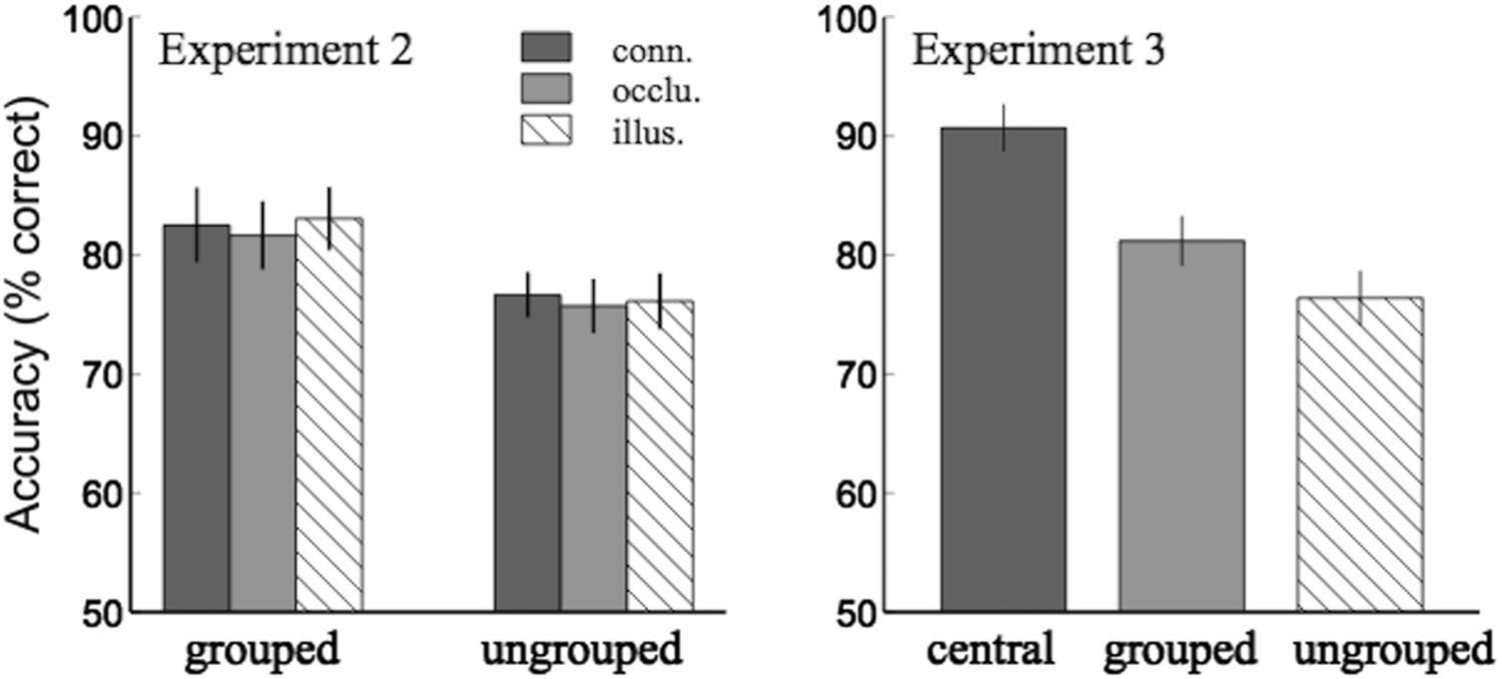
Results of Experiment 2 & 3. Left panel: comparison between the average change detection accuracies for the items inside (grouped) and outside (ungrouped) the Kanizsa triangle in Experiment 2. Right panel: comparison among the accuracies for the central, grouped, and ungrouped items in Experiment 3. Error bars indicated standard errors of the mean.

The results are shown in Fig. 4, the left panel. A 2 × 3 (grouping × configuration) repeated-measures ANOVA was conducted. There was a significant difference between the change detection accuracy for items inside the Kanizsa triangle (grouped) and for those outside (ungrouped), [$F(1,14)=9.90,p=0.007,{\eta }_{p}^{2}=0.41$]. There was no significant difference between the results of the three configuration conditions, [$F(2,28)=1.15,p=0.33,$ ${\eta }_{p}^{2}=0.02$]. No interaction was found, [$F(2,28)=0.15,p=0.86,{\eta }_{p}^{2}=0.01$].

#### Experiment 3

Experiment 2 successfully replicated the grouping effect of connectedness^18^, and extended the effect to situations where items were connected by an occluder or by an illusory figure. This beneficial effect existed even if the tested feature was irrelevant to grouping. However, one might notice that the central item in the memory display always contributed to form the illusory figure, and was always closest to, although not located on, the fixation compared to the other items. Since research showed that space-based selective attention could prioritize visual processing of stimulus near fixation^46,47^ and therefore enhance the memory performance^48^, it was possible that the performance for the central item was better improved than the other grouped items. Could it be attentional selection, not grouping, that accounted for the beneficial effect we observed in Experiment 2? In Experiment 3, we further investigated the grouping effect of illusory contour by disassociating the VWM performance for the central item and the other two grouped items. Both the grouped and ungrouped items formed equilateral triangles to control for a possible confounding factor of general configuration. We predicted that the memory performance for the central item would be better than for the others since it might be prioritized for processing, but the beneficial effect of grouping would still be found for the grouped items.

The results are shown in Fig. 4, the right panel. A repeated-measures ANOVA showed a significant difference among the accuracy for the central item, the grouped items, and the ungrouped items, [$$F(2,28)=33.08,$$$$p < 0.001,{\eta }_{p}^{2}=0.70$$]. Post-hoc comparison showed that the accuracy for the central item was significantly higher than the grouped items, [*F*(1, 14) = 31.17, *p* < 0.001, Bonferroni corrected, $${\eta }_{p}^{2}=0.69$$], and the ungrouped items, [*F*(1, 14) = 47.02, *p* < 0.001, Bonferroni corrected, $${\eta }_{p}^{2}=0.77$$]; and the accuracy for the grouped items was significantly higher than the ungrouped items, [*F*(1, 14) = 9.79, *p* = 0.022, Bonferroni corrected, $${\eta }_{p}^{2}=0.41$$].

The results showed that the change detection accuracy was highest for the central item, supporting our speculation that there was a contribution of spatial proximity in Experiment 2 that memory was improved for items close to or at the fixation. In addition, the accuracy was significantly better for the grouped items than for the ungrouped items, suggesting that grouping effect was robust even after the spatial proximity effect was removed.

## Meta-Analytic Study

Although a number of studies have shown that Gestalt grouping facilitates visual working memory^5,18,37^, a few studies also demonstrated contradictory results^49^. In addition, it remains unclear whether factors like the experimental design, the types of stimuli, or the nature of grouping methods could result in differences in the grouping effect. Therefore, a meta-analytic study was conducted to comprehensively summarize the effect of Gestalt grouping on visual working memory, and to examine the potential moderators that might play a role in the grouping effect. Through this approach, we aimed to resolve controversies raised in past literature, to reveal possible underlying mechanisms involved in the grouping process, and thus to shed lights on future research related to this topic.

### Methods

#### Literature search

To locate the relevant articles, first we conducted online searches of databases in English: psycINFO, Psychology, Web of Sciences, PubMed, Elsevier, Springer, Behavioral Science Collection, ERIC, and Google scholar, using keywords *visual working memory*, *visual short*-*term memory* with combinations of *gestalt*, *perceptional organization*, *grouping*, *similarity*, *proximity*, *connectedness*, *closure*, *collinearity*, *object* and *common fate* between 1997 to 2017. Second, the reference lists of the included articles were scrutinized for studies not indexed in the electronic databases.

#### Criteria for selection of studies

The studies were included if they met the following criteria: (1) the study must be empirical; (2) the participants must be healthy adults with normal or corrected-to-normal vision; (3) the experimental design must include a stimulus display with the memory items organized by at least one Gestalt grouping principle; (4) the outcome variable must be accuracy for memory performance or other measures that can be transformed into accuracy according to a certain formula, e.g. capacity estimation (Cowan’s K), detection sensitivity (*d*′), maximum sensitivity measure (*P*(*c*)_*max*_); (5) the study should provide mean, standard deviation (s.d.), sample size, test statistics or other data that can be calculated for effect size.

The studies were excluded if: 1) they were focused on cognitive performance other than visual working memory (e.g. visual-spatial working memory, visual statistical learning, haptic grouping, Boolean maps); 2) perceptual grouping was achieved using non-Gestalt configurations (e.g. feature binding, within-category similarity, real-world spatial regularity, etc).

**Figure 5:**
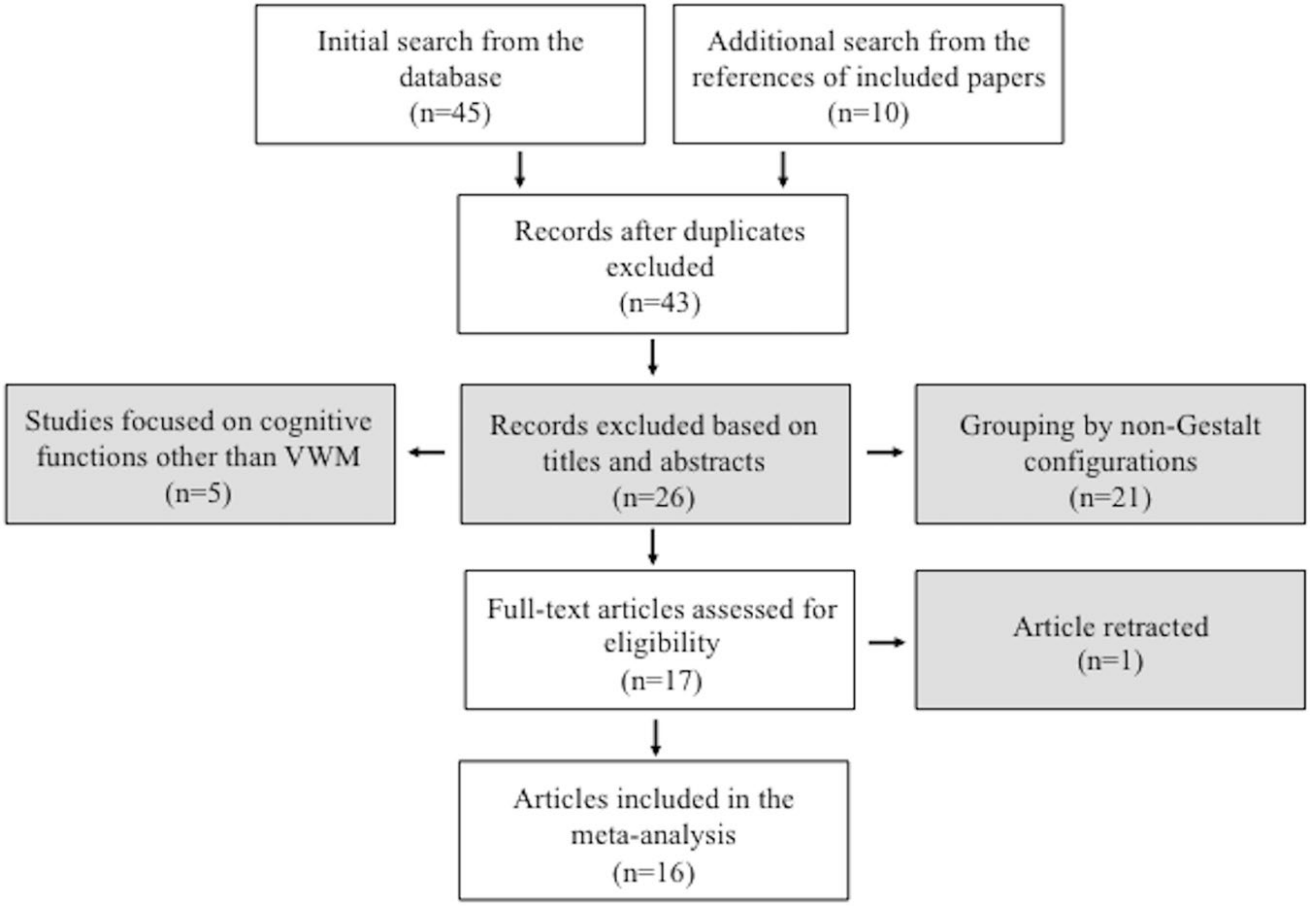
Flow chart of the process in literature selection.

According to the inclusion and exclusion criteria, two of the authors (JL and JQ) judged whether the searched studies should be included in the meta-analysis. We obtained 45 articles from the online database searches and 10 articles from reference searching. After removing the duplicates, we obtained 43 articles, from which 27 were excluded for various reasons (see details in Fig. 5). Sixteen articles fulfilled the selection criteria. The results of Experiment 3 in the present study were also included for analysis. We decided that Experiment 1 and 2 were not eligible for inclusion, since there was a potential confound of grouping by general configuration in the random orientation condition of Experiment 1 and the grouping effect observed in Experiment 2 might be affected by the enhanced performance for one of the grouped items that was always closest to the fixation. Finally, forty-three studies were included in the current meta-analysis, with a total of 961 participants included. The literature search processes were shown in Fig. 5.

#### Coding procedures

To ensure coding reliability, one of the authors (JL) coded the following information of the included studies: 1) basic information on the research, including author(s), publication year, journal (or dissertation); 2) demographic information, including the sample size, age, sex ratio, and the organization in which the participants involved; 3) study characteristics, including the experimental design, types of stimuli, types of memory tasks, independent and dependent variables; and 4) statistical results used for estimating effect sizes, including the mean and s.d. for the experimental conditions, t test, F test or other statistics, and p-values. If certain s.d. was not explicitly reported in the text, it was estimated manually based on the error bars in the corresponding figure, which usually represented standard error or 95% confidence interval (CI).

#### Outcome measures

Research on visual working memory often used accuracy (Acc) of responses or an estimate of capacity as a dependent variable to evaluate VWM performance. Most studies included in this meta-analysis reported the change detection accuracy. Some studies also used one or more of the following measures: (1) estimated capacity of VWM (Cowan’s K), which indicates the maximum number of items one can hold in VWM. A commonly used formula to calculate capacity is *K* = *Set size* × (*Hit* − *False alarm*)^11^. (2) Sensitivity (*d’*) from signal detection theory, which equals *Z*(*Hit*) − *Z*(*False alarm*), where *Z*(*x*) is the inverse of the cumulative probability function of the Gaussian distribution^50^. (3) Maximum sensitivity measure, *P*(*c*)_*max*_, which is an unbiased sensitivity measure corrected for response bias and is linearly related to *d*’^51^. If more than one outcome measures were reported within one sample in an experiment, the measure of accuracy was prioritized for coding since most of the above measures can be computed from accuracy.

#### Moderator variables

Due to the divergence in the experimental design and the stimuli used in the various studies, moderator analysis was necessary to examine the possible factors influencing the grouping effect on VWM. Eight moderators were taken into consideration:

1. Grouping methods. Different grouping methods may yield various effects on VWM. There were 11 grouping methods used in all 43 studies: similarity (memory items shared a same feature, or resembled each other, therefore can be grouped together), connectedness (memory items connected by lines), closure (memory items completed an illusory figure), collinearity (memory items were arranged on a same imaginary line), common fate (memory items moved in the same direction), common region (memory items positioned within a marked region), proximity (memory items positioned close to one another), symmetry (the pattern of the whole memory display was symmetric), whole-part similarity (the shape of a memory item was similar to the shape of the whole display), proximity & similarity, and connectedness & similarity.
2. Nature of the tasks. It is possible that the nature of the experimental paradigm and the tasks used in a study could affect the magnitude of the grouping effect on VWM. Change detection task (CDT) is a commonly used experimental paradigm to examine VWM. In a classic CDT task, memory items are presented simultaneously for a brief duration and then a test display is presented following a retention interval. The test display could contain a probe, alone (single test display) or together with other memory items (whole test display). In a variant of the change detection task, memory items are presented sequentially (sequential CDT). Therefore, the tasks employed in the included studies can be classified into the following categories: CDT with a single test display (CDT/single); CDT with a whole test display (CDT/whole); sequential CDT with a single test display (SCDT/single); and sequential CDT with a whole test display (SCDT/whole).
3. Duration of memory display. Testing the presentation duration of the memory items may have important indications on the underlying mechanisms involved in the process of grouping. In all the studies, the duration of memory display ranged from 17 ms to 2000 ms. Note that for a sequentially displayed task, we added up the presentation time of each memory item as the total duration of the memory display. The studies were classified into the following categories: ≤100 ms, 200–450 ms, 500–600 ms, and ≥1000 ms. Such division was selected since presumably a duration less than 100 ms is considered to be quite short for visual processing, a duration of 200–450 ms is typical for examining VWM, 500–600 ms is less typical but still often being tested, and a duration longer than 1000 ms usually allows compete processing of a visual scene.
4. Feature type. This moderator referred to the type of feature tested in a memory task, and was selected to examine whether certain feature could benefit more from perceptual grouping regardless of its relevancy to grouping formation. Since it was possible that the tested feature was not the feature that was intrinsic to grouping (e.g., in our empirical study, color was the tested feature, but orientation of the circular sectors was the feature that formed perceptual grouping), we differentiated these two factors and performed separate moderator analyses for *feature type* and *grouping relevancy of the tested feature* (see below). Most of the included studies tested the memory performance on one of the three features: color, shape, or orientation. A few tested two features, such as color and shape. The latter studies were classified into one category that termed as ‘dual-features’.
5. Grouping relevancy of the tested feature. Whether the tested feature of a memory item is relevant to grouping might influence its effect on VWM. For example, if memory items were grouped by color similarity, then color is a grouping relevant feature. In this case, testing the memory performance on shape of the items would be considered to be grouping irrelevant. The tested features in the studies were coded as grouping relevant or grouping irrelevant.
6. Verbal suppression task. The studies were also coded for whether the participants were instructed to perform a concurrent articulatory suppression task to prevent verbally encoding and rehearsing the stimuli. This moderator was selected to examine the effect of suppressing verbal encoding and rehearsal for memorizing visual stimuli, which may further indicate whether employing verbal suppression is necessary or redundant when testing VWM.
7. Cue employment. The usage of a cue could introduce the effect of attentional selection in addition to perceptual grouping. Although the formation of perceptual grouping is often considered to be independent of attention^26–28,31^, one cannot rule out the possibility that an explicit manipulation of attention could modulate the effect of grouping. In some of the included studies, a pre-cue or post-cue was displayed to indicate a memory item that was grouped with other items. Therefore, the studies were coded according to cue employment.
8. Presence of competition. Whether the grouped and ungrouped items were simultaneously presented and therefore to induce potential competition for memory resources might influence the grouping effect on VWM. Presenting the grouped and ungrouped items simultaneously indicated presence of competition, whereas presenting them in separate trials indicated absence of competition. Examining this moderator could be informative of the underlying cognitive mechanisms of the grouping benefit. Note that for this moderator analysis, studies employed a sequential change detection task were excluded. Since participants could not identify which item was grouped and which was not in a single presentation of stimuli until the whole sequence of presentations was finished, the process of competition could not be triggered in such a task. Therefore, thirty-four studies that employed a CDT were coded according to presence of competition.

#### Data analysis

The standardized mean difference, Cohen’s *d*^52^, between the memory performance for the grouped and ungrouped conditions was the measure of effect size. Because we predicted that Gestalt grouping principles could improve VWM, indices of effect sizes were given positive signs when the results were in the theoretically predicted direction and negative signs for the opposite. When means and standard deviations were available, we calculated the effect sizes with the formula given by Cohen^52^. This was the case for 34 of the 43 effect sizes (79.1%). As an inferential statistic (typically *t* test, *p*, *r*, or *F*) was available in the remaining cases, the formula provided by Lipsey and Wilson were used^53^. Effect sizes were assessed based on random effects model since the studies varied vastly in their experimental design, grouping methods, nature of stimuli, etc.

In order to ensure the independence of effect sizes, each independent sample could only be coded once and produce one effect size. In other words, study with multiple outcome measures or independent variables other than grouping (e.g. several levels of set size for the memory array) was represented by one synthetic score.

The Stouffer method of adding standard normal deviate *z*s was used for estimating significance levels for combined studies^54^. In addition to computing combined effect sizes and probability levels, for each analysis a fail-safe N was also computed^55^. The fail-safe N is an estimate of the number of unpublished, nonsignificant studies that would have to exist for the obtained probability value to be rendered nonsignificant. Publication bias was also assessed by the trim-and-fill analysis. *Stata 12*.*0* was used to perform the analysis.

### Results

This meta-analysis was conducted on 16 articles, which covered the years 2000 to 2017. Effect sizes were obtained from 43 studies with independent samples, each sample size ranging from 8 to 100. The characteristics of these studies were summarized in Table 1.

**Table 1:** Characteristics of Individual Study.

Study (Author/Year)	Sample size	Set size	Grouping principles	Task (Test display)	Duration (ms)	Stimuli type	Grouping relevancy	Verbal task	Cue	Competition	Outcome measures	Effect size (d)
Balaban (2016)a^83^	15	4	Common fate	CDT (whole)	1100	orient.&color	No	No	Yes	No	Acc	0.11
Balaban (2016)b^83^	15	4	Common fate	CDT (whole)	1100	shape&color	No	No	Yes	No	Acc	0.59
Gao (2015)a^38^	16	4	Collinearity	SCDT (whole)	500	orientation	Yes	Yes	No	No	*d*′	1.88
Gao (2015)b^38^	16	4	Collinearity	SCDT (whole)	500	orientation	Yes	Yes	No	No	*d*′	0.93
Gao (2015)c^38^	16	3	Closure	SCDT (whole)	450	orientation	Yes	Yes	No	No	*d*′	1.05
Gao (2015)d^38^	16	4	Collinearity	SCDT (single)	500	orientation	Yes	Yes	No	No	*d*′	0.82
Gao (2015)e^38^	16	4	Collinearity	SCDT (single)	500	orient.&color	Yes	Yes	Yes	No	*d*′	0.60
Gao (2015)f^38^	22	6	Similarity	SCDT (single)	400	color	Yes	Yes	No	Yes	*d*′	0.34
Kalamala (2017)a^84^	42	9	Symmetry	CDT (whole)	2000	shape	No	No	No	No	K	0.08
Kalamala (2017)b^84^	100	5, 6	Symmetry	CDT (whole)	2000	shape	No	No	No	No	K	0.07
Kalamala (2017)c^84^	60	5, 6	Whole-part simil.	CDT (whole)	2000	shape	No	No	No	No	K	0.24
Kalamala (2017)d^84^	41	3, 4, 5	Whole-part simil.	CDT (whole)	2000	shape	No	No	No	No	K	0.31
Lamsweerde (2016)a^85^	26	6	Connectedness	CDT (whole)	500	color	Yes	No	No	No	Acc	0.13
Lamsweerde (2016)b^85^	22	6	Similarity	CDT (whole)	500	shape	Yes	No	No	Yes	Acc	0.43
Lamsweerde (2016)c^85^	21	6	Similarity	CDT (whole)	500	shape&color	Yes	No	No	Yes	Acc	0.52
Lin (2009)a^86^	20	3, 4	Similarity	CDT (single)	200	color	Yes	Yes	No	No	Acc	1.78
Lin (2009)b^86^	18	3	Similarity	SCDT (single)	600	color	Yes	Yes	No	No	Acc	1.66
Lin (2009)c^86^	20	3	Similarity	SCDT (single)	600	color	Yes	Yes	No	No	Acc	1.92
Lin (2009)d^86^	20	3	Similarity	SCDT (single)	600	color	Yes	Yes	No	Yes	Acc	1.03
Luria (2014)a^87^	16	8	Common fate	CDT (whole)	100	color	No	No	Yes	No	Acc	0.16
Luria (2014)b^87^	16	8	Common fate	CDT (whole)	600	color	No	No	Yes	No	Acc	0.47
Luria (2014)c^87^	16	8	Common fate	CDT (whole)	17	color	No	No	Yes	No	Acc	0.25
McCollough (2011)^88^	16	3	Closure	CDT (single)	500	orientation	Yes	No	No	No	Acc	1.38
Morey (2015)^89^	57	3, 5, 7	Similarity	CDT (single)	1200	color	Yes	Yes	No	Yes	Acc	0.38
Neira (2016)^90^	16	2, 4, 6	Closure	CDT (whole)	250	orientation	Yes	No	No	No	Acc	0.63
Peterson (2013)a^5^	10	3, 4, 6	Similarity	CDT (single)	200	color	Yes	No	No	Yes	Acc, K	4.46
Peterson (2013)b^5^	13	6	Proxi. + simil.	CDT (single)	200	color	Yes	No	No	Yes	Acc, K	1.09
Peterson (2015)a^49^	22	6	Proxi. + simil.	CDT (single)	100	color	Yes	No	Yes	Yes	Acc	1.70
Peterson (2015)b^49^	15	6	Connectedness	CDT (single)	100	color	No	No	Yes	Yes	Acc	1.09
Peterson (2015)c^49^	19	6	Conn. + simil.	CDT (single)	100	color	Yes	No	Yes	Yes	Acc	−0.81
Quinlan (2012)a^91^	30	6	Similarity	CDT (single)	250	shape&color	Yes	No	No	Yes	*P*(*c*)_*max*_	1.22
Quinlan (2012)b^91^	30	6	Similarity	CDT (single)	250	shape&color	Yes	No	No	Yes	*P*(*c*)_*max*_	1.66
Xu (2007)a^37^	12	3	Common region	CDT (single)	200	shape	No	No	No	Yes	K	1.74
Xu (2007)b^37^	8	2	Common region	CDT (single)	200	shape	No	No	No	No	K	0.19
Xu (2012)a^92^	16	4	Similarity	CDT (single)	100	color	Yes	No	Yes	No	Acc	4.08
Xu (2012)b^92^	23	3	Similarity	CDT (single)	100	color	Yes	No	Yes	Yes	Acc	1.49
Xu (2012)c^92^	17	3	Similarity	CDT (single)	100	color	Yes	No	Yes	Yes	Acc	1.88
Woodman (2003)a^18^	12	6	Proximity	CDT (whole)	100	color	No	Yes	Yes	Yes	Acc	1.74
Woodman (2003)b^18^	12	4, 6	Connectedness	CDT (whole)	100	color	No	Yes	Yes	No	Acc	1.09
Woodman (2003)c^18^	12	6	Proximity	CDT (whole)	100	color	No	Yes	Yes	Yes	Acc	1.19
Zhang (2015)a^93^	20	3, 4	Similarity	CDT (whole)	350	orientation	Yes	Yes	No	No	Acc	0.49
Zhang (2015)b^93^	16	2, 3, 4	Similarity	CDT (whole)	350	orientation	Yes	Yes	Yes	No	Acc	2.90
Our study (Exp.3)	15	5	Closure	CDT (whole)	250	color	No	No	No	Yes	Acc	0.59

#### The overall grouping effect on VWM

**Figure 6:**
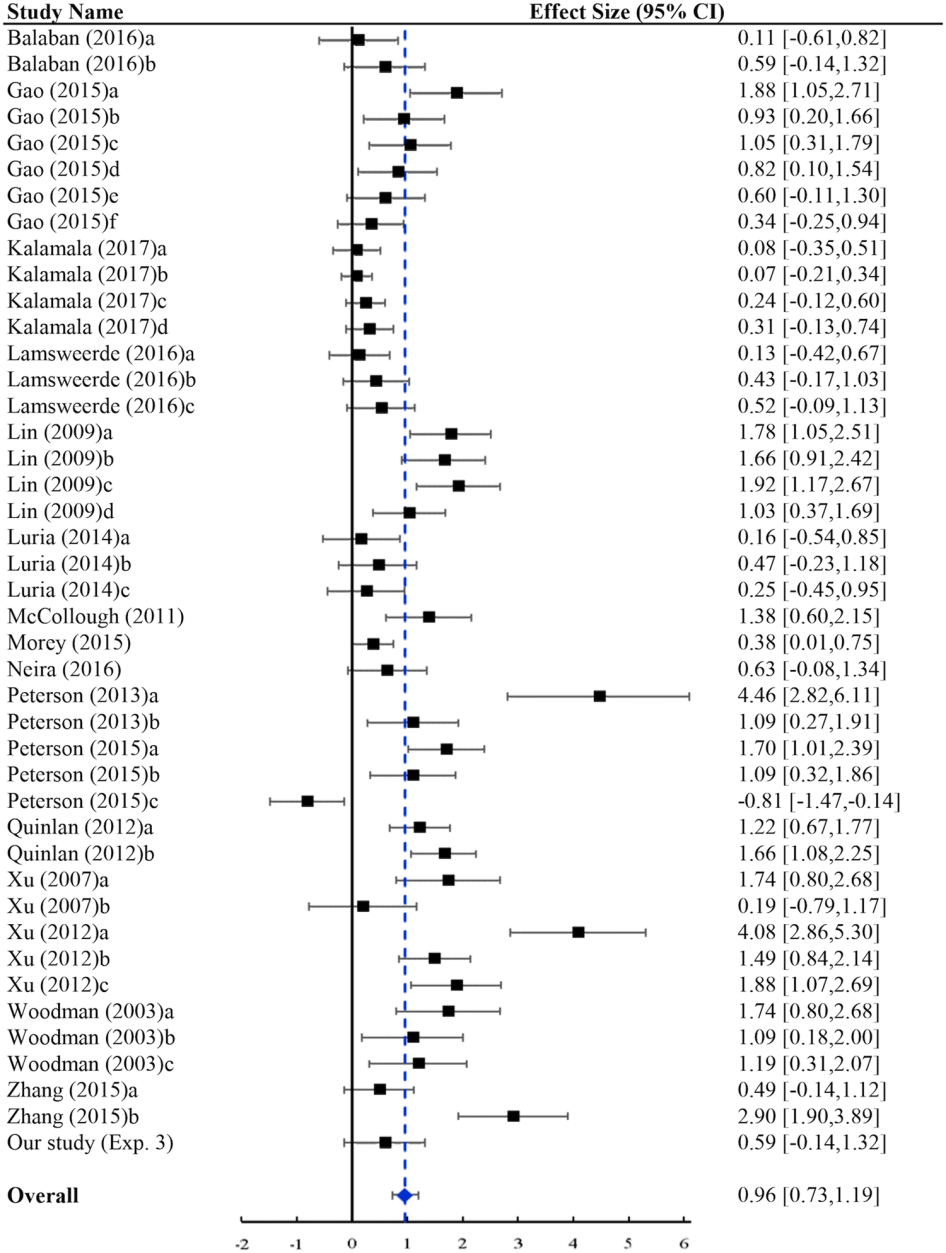
Overall effect of grouping on visual working memory. For the individual studies, the size of the square indicated the weight for the effect size in each study, and the line indicated the 95% CI of each effect size; for the overall effect, the width of the diamond indicated the 95% CI of the overall effect size, and the line indicated its 95% prediction interval.

Figure 6 presents the forest plot of the overall grouping effect on visual working memory. Results of this analysis revealed a mean estimated Cohen’s *d* of 0.96 with 95% CI = 0.73–1.19, which could be considered a fairly large effect^52^. When translated into *U*_3_ metrics^55^, this amount of *d* suggested that the capacity of VWM could be improved by an average of 33.2%, i.e., from the putative limit of 4 items to 5.33 items. Stouffer method of significance testing showed that grouping significantly improved memory performance, *p* < 0.001 (Table 2). The test of heterogeneity was significant, *Q* = 230.54, *P* < 0.001, suggesting that the effect sizes varied significantly across different studies. Examining the potential moderators might therefore clarify the factors that account for this variability. A file drawer analysis or fail-safe N indicated that 1009 missing or nonpublished studies that averaged null findings would have to exist to cancel this effect. However, the existence of so many studies was unlikely given the number of the included studies in the current meta-analysis.

**Table 2:** Main results.

Analysis	No. of studies	Sample size	Effect size *d* (95%*CI*)	Heterogeneity			Combined *Z*	*p*	Fail-safe *N*
				*Q*	*p*	*I* ^2^			
**Overall**	43	961	0.96 [0.73, 1.19]	230.54	<0.001	81.8	8.14	<0.001	1009
**Moderator analysis**									
*Gestalt grouping*									
Similarity	16	362	1.49 [1.05, 1.93]	102.86	<0.001	85.4	6.59	<0.001	240
Common fate	5	78	0.31 [−0.01, 0.63]	1.29	0.862	<0.1	1.93	0.054	—
Closure	4	63	0.89 [0.52, 1.26]	2.88	0.411	<0.1	4.76	<0.001	29
Collinearity	4	64	1.03 [0.51, 1.54]	5.83	0.120	48.5	3.88	<0.001	18
Connectedness	3	53	0.71 [0.00, 1.41]	5.58	0.061	64.2	1.97	0.049	1
Dual effects	3	54	0.66 [−0.92, 2.23]	28.49	<0.001	93.0	0.82	0.414	—
Common region	2	20	0.97 [−0.55, 2.50]	5.02	0.025	80.1	1.25	0.211	—
Proximity	2	24	1.45 [0.80, 2.09]	0.70	0.401	<0.1	4.41	<0.001	12
Symmetry	2	142	0.07 [−0.16, 0.30]	<0.01	0.956	<0.1	0.59	0.552	—
Whole-part similarity	2	101	0.27 [−0.01, 0.55]	0.06	0.813	<0.1	1.90	0.057	—
*Task*									
CDT (single)	15	308	1.44 [0.93, 1.95]	105.84	<0.001	86.8	5.53	<0.001	154
CDT (whole)	19	493	0.52 [0.29, 0.74]	49.21	<0.001	63.4	4.46	<0.001	120
SCDT (single)	6	112	1.04 [0.55, 1.53]	14.93	0.011	66.5	4.15	<0.001	32
SCDT (whole)	3	48	1.26 [0.69, 1.82]	3.23	0.199	38.0	4.38	<0.001	18
*Duration*									
≤100 *ms*	11	180	1.21 [0.55, 1.86]	76.72	<0.001	87.0	3.62	<0.001	42
200–450 *ms*	13	228	1.26 [0.82, 1.71]	54.14	<0.001	77.8	5.55	<0.001	134
500–600 *ms*	12	223	0.94 [0.61, 1.28]	31.63	<0.001	65.2	5.47	<0.001	120
≥1000 *ms*	7	330	0.21 [0.06, 0.36]	3.53	0.740	<0.1	2.66	<0.001	11
*Stimuli type*									
Color	22	417	1.15 [0.76, 1.53]	133.04	<0.001	84.2	5.85	<0.001	256
Orientation	8	132	1.20 [0.72, 1.68]	22.56	0.002	69.0	91	<0.001	63
Shape	7	285	0.30 [0.04, 0.56]	12.33	0.055	51.3	2.23	0.026	5
Dual-features	6	127	0.81 [0.35, 1.27]	15.34	0.009	67.4	3.44	0.001	20
*Grouping relevancy*									
Yes	27	554	1.21 [0.89, 1.53]	154.66	<0.001	83.2	7.36	<0.001	513
No	16	407	0.50 [0.27, 0.74]	34.27	0.003	56.2	4.22	<0.001	89
*Verbal task*									
Yes	16	309	1.18 [0.85, 1.52]	53.41	<0.001	71.9	6.90	<0.001	265
No	27	652	0.83 [0.53, 1.13]	159.40	<0.001	83.7	5.49	<0.001	273
*Cue*									
Yes	16	258	1.10 [0.60, 1.60]	100.20	<0.001	85.0	4.32	<0.001	94
No	27	703	0.88 [0.63, 1.13]	123.80	<0.001	79.0	6.87	<0.001	443
*Competition*									
Yes	16	330	1.16 [0.75, 1.56]	84.02	<0.001	82.1	4.22	<0.001	89
No	18	471	0.71 [0.38, 1.05]	92.97	<0.001	81.7	5.53	<0.001	185

#### Moderator analyses

The results of moderator analyses were presented in Table 2. Overall, we found that the grouping principles used in the study, the nature of the tasks performed, the presentation duration of memory items, the probing manner, and the characteristics of stimuli could influence the effect of grouping, whereas the employment of cue or a verbal suppression task could not.

*Grouping methods*. Eleven different grouping methods were employed in the included studies. Most studies used one Gestalt principle to examine the grouping effect, except for three studies that used two Gestalt principles, such as, proximity & similarity^5,49^ and connectedness & similarity^49^. Since these three studies were conducted by the same research team, the three effect sizes estimated from these studies were categorized into a single subgroup termed as ‘dual effects’. Therefore, we obtained ten subgroups in total, each employed one grouping methods. The test of heterogeneity was significant, *Q* = 77.98, *p* < 0.001, suggesting that variance differed significantly across different subgroups.

**Figure 7:**
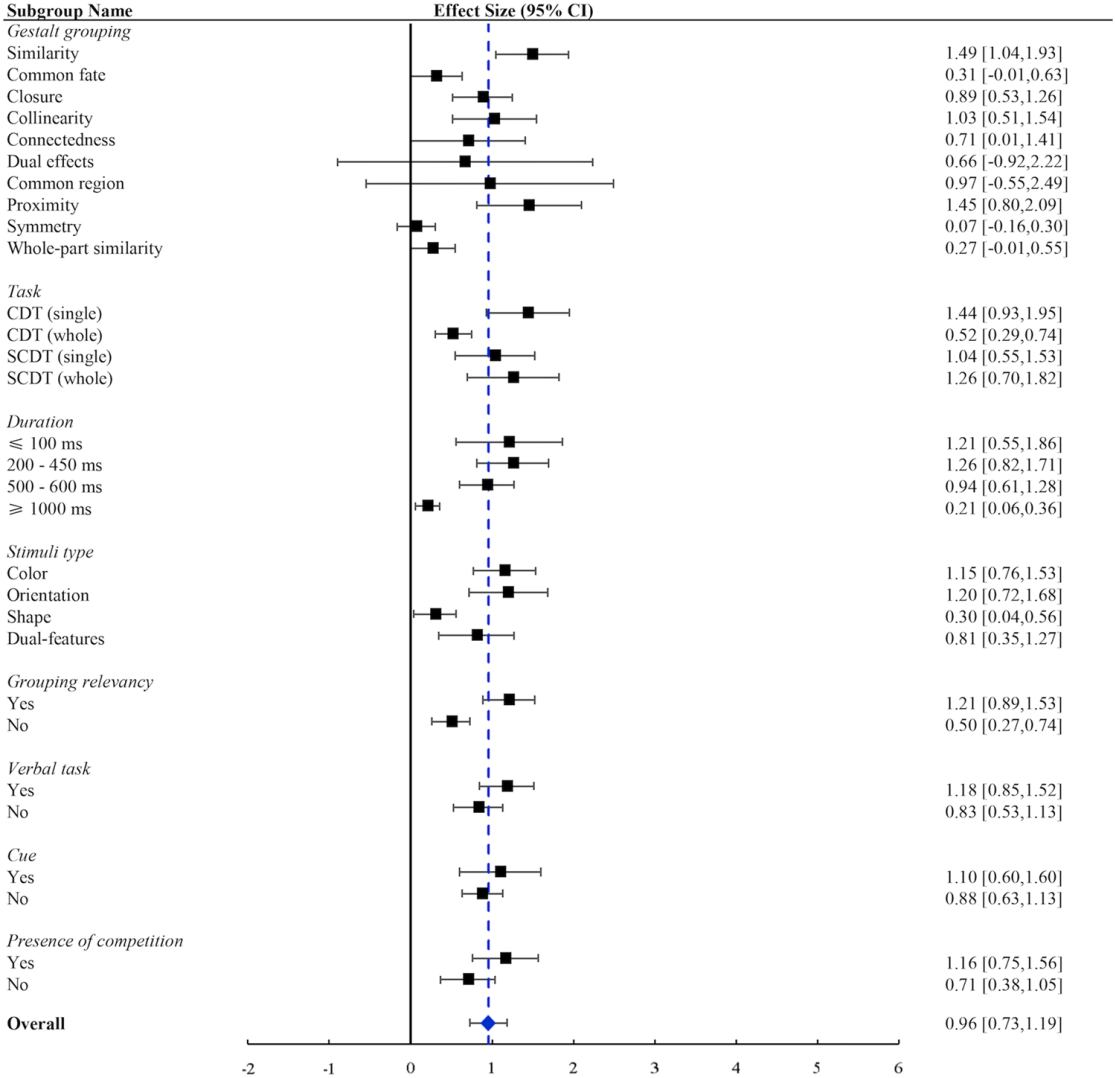
Forest plots for all moderators.

The results showed that grouping by closure, collinearity, connectedness, proximity or similarity significantly enhanced VWM (Table 2, also see Fig. 7). The effects of these grouping methods were: similarity, *d* = 1.49 (*p* < 0.001); proximity, *d* = 1.45 (*p* < 0.001); collinearity, *d* = 1.03 (*p* < 0.001); closure, *d* = 0.89 (*p* < 0.001); and connectedness, *d* = 0.71 (*p* = 0.049). This suggests that the beneficial effect does differ across different grouping methods. Among these subgroups, Q-tests showed that the grouping effects were only significantly different between similarity and closure (*Q* = 4.18, *p* = 0.041). The homogeneity tests were not significant except for similarity, suggesting that the variability within the other four subgroups was possibly due to sampling errors (Table 2). However, failure to reject homogeneity might also be due to a lack of power since there were only 2 to 4 studies in these subgroups. The results of the homogeneity test suggested that unidentified factors might account for the variance in the subgroup of similarity, and we suspected that similarity grouping by different features might contribute to at least part of heterogeneity. In addition, file-drawer analysis showed that 240 missing studies with an averaged null effect would have to exist to cancel the effect of similarity grouping, 29 for closure grouping, 18 for collinearity, 12 for proximity, and 1 for connectedness. This suggests that the results were relatively robust for the former four grouping methods, but not that reliable for connectedness grouping.

For the other subgroups of grouping methods, the effect of grouping was not significant: common fate (*d* = 0.31, *p* = 0.054), dual effects (*d* = 0.66, *p* = 0.414), common region (*d* = 0.97, *p* = 0.211), symmetry (*d* = 0.07, *p* = 0.552), and whole-part similarity (d = 0.27, 0.057). This suggested that these five grouping methods might not be as effective as the other methods listed above. Since the main grouping effect was not significant, Q-test comparisons were not performed for these subgroups. The homogeneity tests showed that there were other variances besides sampling errors within the subgroups of dual effects and common region. Based on the characteristics of the individual studies reported in Table 1, we suspected that the different combinations of the grouping methods might contribute to at least part of heterogeneity in the subgroup of dual effects, and the presence of competition might be a source of variability in the subgroup of common region.

*Nature of the tasks*. The included studies were classified into five subgroups according to the nature of the tasks and the homogeneity test showed that the between-subgroup variance was significant (*Q* = 55.72, *p* < 0.001), indicating that this moderator successfully explained at least some of the variability among the studies.

Among all of the included experimental paradigms, the grouping effect found in the CDT/single task was the strongest (*d* = 1.44, *p* < 0.001), followed by SCDT/whole (*d* = 1.26, *p* < 0.001), SCDT/single (*d* = 1.04, *p* < 0.001), and CDT/whole (*d* = 0.52, *p* < 0.001), see Fig. 7. This suggests that the nature of the tasks does affect the measured magnitude of grouping effect. The Q-tests demonstrated that there were significant differences between the grouping effect in a CDT/single task and that in a CDT/whole task (*Q* = 10.46, *p* = 0.001), and SCDT/whole and CDT/whole task (*Q* = 5.69, *p* = 0.016). The homogeneity tests for CDT/single, CDT/whole, and SCDT/single were significant, suggesting that unidentified factors might account for the variance in these subgroups (Table 2). However, failure to reject homogeneity for the SCDT/whole subgroups might be due to a lack of power since there were only 3 studies in this subgroup, therefore we should treat these results with caution. File-drawer analysis showed that fail-safe N was 120 for a CDT/whole task, 154 for a CDT/single task, 32 for a SCDT/single task, and 18 for a SCDT/whole task. Again, the existence of such a large pool of missing studies seem to be unlikely.

*Duration of memory display*. The included studies were classified into four subgroups and the homogeneity test showed that the between-subgroup variance was significant (*Q* = 65.04, *p* < 0.001). The grouping effect was strongest if the duration was between 200 ms and 450 ms (*d* = 1.26, *p* < 0.001), followed by a duration of less than or equal to 100 ms (*d* = 1.21, *p* < 0.001), 500–600 ms (*d* = 0.94, *p* < 0.001), and that of greater than or equal to 1000 ms (*d* = 0.21, *p* = 0.007), see Fig. 7. The Q-test showed that the effect sizes of the former three subgroups were all significantly larger than the subgroup of ≥1000 *ms* (≤100 *ms*, *Q* = 8.51, *p* = 0.004; 200–450 *ms*, *Q* = 19.21, *p* < 0.001; 500–600 *ms*, *Q* = 15.19, *p* < 0.001). Overall, the grouping effect on VWM was quite large when memory array was presented for less than 500 ms. File-drawer analysis showed that fail-safe N was 42, 134, 120, and 11 for a duration of ≤100 *ms*, 200–450 *ms*, 500–600 *ms*, and ≥1000 *ms*, respectively.

*Feature type*. The included studies were classified into four subgroups and the homogeneity test showed that the between-subgroup variance was significant (*Q* = 47.79, *p* < 0.001). The grouping effect was strongest if orientation was tested (*d* = 1.20, *p* < 0.001), followed by color (*d* = 1.15, *p* < 0.001), dual-features (*d* = 0.81, *p* = 0.001), and shape (*d* = 0.30, *p* = 0.026), see Fig. 7. The Q-test showed that grouping effect was greater for orientation than shape (*Q* = 10.44, *p* < 0.001), and for color than shape (*Q* = 12.85, *p* < 0.001). File-drawer analysis showed that fail-safe N was 256, 63, 5, and 20 for color, orientation, shape and dual-features, respectively. The small fail-safe N for shape indicated that this result should be treated with caution.

*Grouping relevancy of the tested feature*. Twenty-seven studies tested features that was crucially relevant to forming the perceptual group, and the other sixteen studies tested on grouping irrelevant features. The homogeneity test showed that the between-subgroup variance was significant (*Q* = 41.76, *p* < 0.001). If the tested feature was grouping relevant, the effect size was large (*d* = 1.21, *p* < 0.001); if not, the effect size was moderate (*d* = 0.50, *p* < 0.001), see Fig. 7. The Q-test showed that VWM was significantly enhanced when the tested feature was directly related to grouping compared to that was unrelated (*Q* = 12.29, *p* < 0.001). File-drawer analysis showed that fail-safe N was 513 for grouping relevant features and 89 for grouping irrelevant features.

*Verbal task*. Sixteen studies employed an articulatory suppression task to minimize the contributions from verbal working memory, and the other 27 did not. The homogeneity test showed that the between-subgroup variance was significant (*Q* = 17.55, *p* < 0.001). The effect sizes for employing a verbal suppression task was *d* = 1.18 (*p* < 0.001), and for not employing such a task was *d* = 0.83 (*p* < 0.001), see Fig. 7. The result of Q-test showed no significant difference in the grouping effect between the two subgroups (*Q* = 2.33, *p* = 0.127). File-drawer analysis showed that fail-safe N was 265 and 273 for employing and not employing a verbal task, respectively.

*Cue employment*. Sixteen studies employed a pre-cue or post-cue to examine the grouping effect on VWM, and the others did not. The homogeneity test showed that the between-subgroup variance was significant (*Q* = 6.68, *p* = 0.010). The effect sizes for employing a cue was *d* = 1.10 (*p* < 0.001), and for not employing a cue was *d* = 0.88 (*p* < 0.001), see Fig. 7. The results of the Q-test showed that the effect sizes did not significantly differ between the two subgroups (*Q* = 0.59, *p* = 0.441). File-drawer analysis showed that fail-safe N was 94 and 443 for with and without a cue employment, respectively.

*Presence of competition*. The grouped and ungrouped items were simultaneously presented in 16 out of 34 studies, suggesting presence of competition. The homogeneity test showed that the between-subgroup variance was significant (*Q* = 23.37, *p* < 0.001). The effect size for competition present was large (*d* = 1.16, *p* < 0.001), and for competition absent was moderate (*d* = 0.71, *p* < 0.001), see Fig. 7. The results of the Q-test showed that the difference in effect size between the two subgroups was marginally significant (*Q* = 2.82, *p* = 0.093). File-drawer analysis showed that fail-safe N was 89 and 185 for presence and absence of competition, respectively.

In addition, we performed a subgroup analysis for competition present and absent condition based on the grouping relevancy of the tested feature. Among eighteen studies that tested a grouping relevant feature (studies used a SCDT were excluded), eleven studies employed a memory display without presence of competition (Table 3). The effect sizes for a competition-present memory display (*d* = 1.13, *p* < 0.001) and a competition-absent display (*d* = 1.54, *p* = 0.001) were large. The results of the Q-test showed that the effect sizes did not significantly differ between the two subgroups (*Q* = 0.61, *p* = 0.433). File-drawer analysis showed that fail-safe N was 58 and 23 for presence and absence of competition under this condition, respectively. On the other hand, among sixteen studies that tested a grouping irrelevant feature, eleven studies employed a memory display with presence of competition. The effect size for a competition-present display was large (*d* = 1.21, *p* < 0.001), but for a competition-absent display was very small (*d* = 0.22, *p* = 0.005). The results of the Q-test showed that VWM for grouping irrelevant features was significantly enhanced when there was presence of competition (*Q* = 17.78, *p* < 0.001). File-drawer analysis showed that fail-safe N was 49 and 20 for presence and absence of competition under this condition, respectively.

**Table 3:** Subgroup analysis.

Analysis	No. of studies	Sample size	Effect size *d* (95%*CI*)	Heterogeneity			Combined *Z*	*p*	Fail-safe *N*
				*Q*	*p*	*I* ^2^			
**Grouping relevant**									
*Competition*									
present	11	264	1.13 [0.60, 1.67]	76.73	<0.001	87.0	4.14	<0.001	58
absent	7	130	1.54 [0.67, 2.42]	56.77	<0.001	89.4	3.46	0.001	23
**Grouping irrelevant**									
*Competition*									
present	5	66	1.21 [0.77, 1.64]	5.29	0.259	24.4	5.45	<0.001	49
absent	11	341	0.22 [0.07, 0.37]	6.84	0.741	<0.1	2.80	0.005	20

#### Publication bias

**Figure 8:**
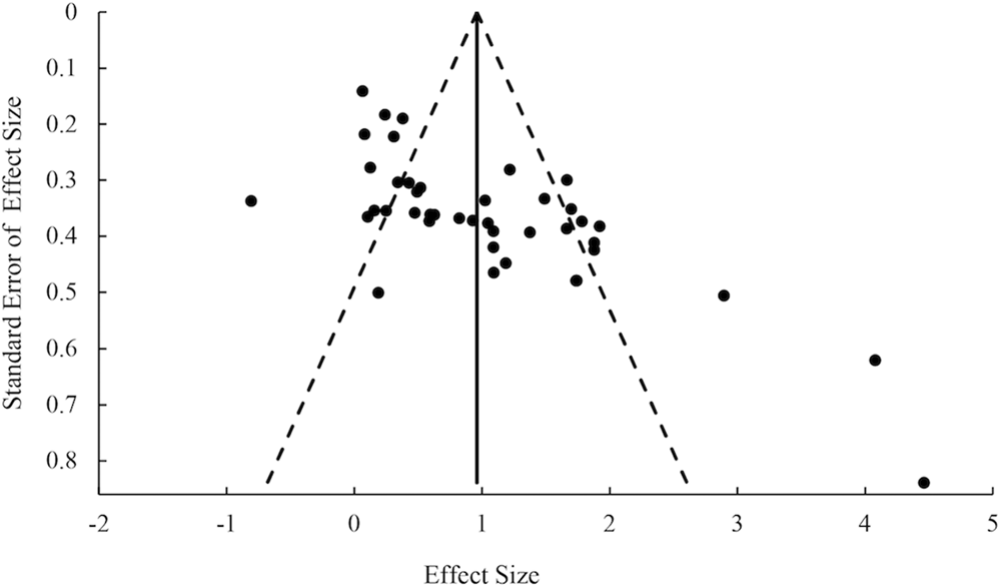
Funnel plot.

In addition to the file-drawer analysis, a trim-and-fill analysis was performed to assess the publication bias. According to the results of Duval and Tweedie’s trim-and-fill, no study should be trimmed using random-effects model. The distribution of effect sizes was shown in the funnel plot (Fig. 8). Majority of effect sizes were well balanced around the mean effect size, except for two studies that had very large effects (*d* > 4.0). One study had relatively small sample size (10 compared to an average of 23), we suspected that the exceedingly large effect size might result from individual differences and a large sampling error. Overall, the funnel plot indicated that most of the selected studies had appropriate sample size and relatively good quality, and we think that the publication bias was negligible in the present meta-analysis.

## Discussion

This research presents an empirical study that investigated the effect of grouping by illusory contour on VWM and a meta-analytic study that summarized the effect of grouping by different Gestalt principles. In our experiments, circular sectors were arranged in a way to produce the Kanizsa illusion. By inducing a percept of an illusory figure occluding the disks, circular sectors were perceived to be grouped by Gestalt principle of closure. Experiment 1 shows that the change detection accuracy is not significantly different between the items that formed a Kanizsa illusion and items that did not, and two possible explanations may account for the lack of a significant effect; Experiment 2 shows that the memory performance is enhanced for the grouped items, and there is no significant difference between grouping by physical connectedness and by closure induced from illusory contour; Experiment 3 shows that the beneficial effect of grouping persists even after partialling out the contributions of spatial proximity effect. In our meta-analysis, we found that the beneficial effect of grouping on VWM is robust, and the magnitude of the effect can be affected by several moderators.

In our empirical study, Experiment 1 employed a same experimental design as in Experiment 2 of Gao *et al*.’s study^38^, except that our task was to detect changes in color instead of orientation and the memory items were presented simultaneously instead of sequentially. However, unlike Gao *et al*. who found a significant improvement in the memory performance for the orientation of the grouped items, our results did not show a significant difference in memory performance between the three grouping configuration conditions and the random orientation condition. Since orientation is a feature critical for grouping formation whereas color is a grouping irrelevant feature, it is possible that this distinction affect the grouping effectiveness. However, the lack of a significant effect may also result from two other possible reasons. First, the general configuration of the memory display might dominate the perception since the items were located at the vertices of a triangle/square/polygon in all four configuration conditions. Indeed, research shows that the construction of a perceptual whole reduces awareness of detailed differences in the parts of the whole object^56^, and Allon *et al*. found that memory performance for stimuli forming a configuration of simple geometric figure was improved compared to others (the ‘proximity grouping’ condition)^57^. Therefore, grouping by general configuration might occur in all four conditions and the visual system ignored the detailed differences such as whether the sectors were physically connected or not. As a result, memory performance was improved in all four conditions and no significant effect was observed. Second, the grouping effect on VWM may involve a process of competing for memory resources between the grouped and ungrouped items. Although perceptual grouping seems to occur at an early stage of visual processing independent of attention^26–28,31^, it may induce to a biased allocation of attention or memory resources toward the grouped items over the ungrouped items after forming a perceptual group. Consequently, the grouped item may be prioritized for processing and receive more resources for memorizing than the ungrouped items. Since the grouped and ungrouped items were tested in separate conditions in Experiment 1, the competition process was not triggered and therefore no significant effect was observed. In Experiment 2, as we presented the grouped and ungrouped items simultaneously within one memory display and avoided a general configuration, the grouping benefit of the illusory contour showed. This suggests that grouping by illusory contour (closure) significantly improves visual working memory, and the beneficial effect exists even if the tested feature is irrelevant to grouping. In addition, we found that the magnitude of the grouping effect is equivalent among illusory contour, physical connectedness and solid occlusion. This suggests that perceiving illusory contours and real lines may share a similar underlying mechanism, consistent with the physiological findings that cells in the visual cortex of monkeys respond to illusory contours as if they were formed by real lines or edges^58^.

In Experiment 3, we found an average of 6% increase in accuracy between the grouped and ungrouped items, which is consistent with the previously reported 6% improvement in memory performance when items were grouped by connectedness^18^. However, since both of the grouped items and the ungrouped items could constitute a configuration of triangle with the central item, one might suspect that the memory performance for the ungrouped items was also improved due to grouping by configuration. In this case, grouping by illusory contour may in fact have a greater beneficial effect on VWM than that is observed in Experiment 3 due to a possible performance elevation in the ungrouped condition. Although we cannot completely rule out this possibility, it seems unlikely that the central item can be simultaneously grouped with the two items that formed a Kanizsa triangle and with the two randomly oriented items. Most studies on perceptual organization indicate that an item could only belong to one perceptual group at a time^18,20,59^ and so far no evidence showed that an item could be simultaneously perceived to belong to more than one group. If the central item is perceived to belong to the Kanizsa figure, it may be naturally excluded from grouping with the other items. Therefore, we think that the grouping effect reported in Experiment 3 involves minimal influence of grouping by general configuration.

The perception of the central item seems to be crucial in inducing the grouping effect in Experiment 3 as the visual system determines which of items should group together based on its orientation. It is possible that the central item is processed prior to the other items, it receives more attention and memory recourses and therefore yields better memory performance than the others. Since the configuration of the items in the grouped and ungrouped conditions was otherwise equivalent except for their orientations in relation to the central item, we speculate that grouping with the central item may result in a percept similar to increasing the visual salience of the grouped items (although Luria *et al*. suggest in a review that the mechanism of grouping might be more complex than that of salience^60^). Previous studies show that visual salience could benefit various perceptual performance and working memory performance by improving the bottom-up distinctiveness of an item and consequently biasing attention to prioritize its visual processing^61–66^. Similarly in our study, the improved performance for the grouped item suggest that these items may be prioritized for attentional selection due to perceptual grouping and their memory performance is facilitated even when the tested feature is grouping irrelevant. In other words, bottom-up attention may be driven by perceptual grouping and the beneficial effect of grouping may reflect a ‘prior entry’ of grouped items, which we would further discuss in the following sections.

### Implications from the meta-analysis

Pulling from the results of 43 studies with a total of 961 participants, we found that perceptual grouping reliably facilitates VWM, but the effect differs among various Gestalt principles. In addition, experimental settings like the nature of the task, the duration of memory display, the characteristics of the tested feature, etc., could moderate the magnitude of the grouping effect.

Grouping by similarity, proximity and collinearity demonstrates strong beneficial effects on VWM. The relatively large fail-safe Ns suggest that the effects cannot possibly be due to publication bias. The effect sizes for grouping by similarity and proximity were approximately the same. Although there is a study showing that similarity grouping might rely on proximity to work^5^, our results suggest that the effect of similarity grouping does not depend on proximity. In line with our findings, there are studies showing that the effect of similarity grouping (color, shape, luminance) is equivalent to that of proximity grouping^23^, and may even override proximity grouping under certain conditions^21,23^. In addition, we found that simultaneously applying two grouping methods does not result in an additive effect. The effect size for the ‘dual effects’ (two out of three studies used proximity and similarity grouping) is moderate but not significant. Specifically, it is smaller than either applying similarity grouping or proximity grouping alone. This suggests that the grouping effects of similarity and proximity cannot be simply accumulated, and may even interfere with each other. Closure and connectedness also demonstrate a moderate grouping effect, however, file drawer analysis suggests that the effect of connectedness is not as reliable as the other grouping methods. On the other hand, grouping by common fate, common region, symmetry, or whole-part similarity does not show a significant beneficial effect on VWM. But note that the number of studies applying these grouping methods is quite small, thus there may not be enough evidence for a conclusive statement.

The nature of the experimental tasks used in a study also seems to affect the magnitude of the grouping effect on VWM. In particular, using CDT with a whole test display (*d* = 0.52) yields much less grouping effect than using the other tasks. This seems to be counterintuitive, as research shows that during the test phase, presenting the probe along with the other memory items results in better memory performance than presenting a single probe^67^. However, when the characteristics of this set of studies were cross-referenced, we found that there was a significant confounding factor influencing its results. Ten out of the 19 studies using a CDT and a whole test display coincide with those studies that tested a grouping irrelevant feature with a competition-absent memory display (GIR/CA), and the latter has a mean effect size of 0.22. In other words, the CDT/whole subgroup almost exclusively incorporates the GIR/CA studies (10 out of 11 in total), and therefore its effect size is strongly biased by this factor. Removing the GIR/CA studies from the the CDT/whole subgroup produces a effect size of 0.87 ± 0.46, and this effect size is not significantly different from the effect sizes for the other tasks (CDT/single, *Q* = 3.40, *p* = 0.065; SCDT/whole, *Q* = 0.52, *p* = 0.472; SCDT/single, *Q* = 0.59, *p* = 0.441). Therefore, the results show that by excluding a prominent confounding factor, the type of experimental tasks examined in our meta-analysis does not significantly moderate the grouping effect on VWM.

The duration of memory display demonstrates an intriguing pattern with regard to moderating the effect of grouping on VWM. The effect size for a duration less than 100 ms was 1.21 ± 0.33, and it increased by a small amount to 1.26 ± 0.45 for a duration between 200–450 ms, then decreased to 0.94 ± 0.17 for a duration between 500–600 ms, and finally diminished to 0.21 ± 0.08 for a duration longer than 1000 ms. As far as we know, this pattern has not been reported in the past literature and it is unclear why the specific variation on the effect size occurs. It is possible that a duration less than 100 ms is not sufficient for effective processing of the whole display as well as the grouping pattern, therefore its effect size is smaller than that for a duration of 200–450 ms. Research shows that the processing time for grouping by proximity and collinearity requires about 88 and 119 ms, respectively, for processing to be completed^68^, and it may take even longer for grouping based on other principles like closure, common fate, etc. As the duration increases, perceptual grouping gradually completes and the effect size increases until it reaches the maximal effect. However, as the duration further increases, additional time for better encoding and storing the ungrouped items becomes available and the memory performance for the ungrouped items might gradually catch up with that for the grouped items, therefore the effect size begins to decrease. With a duration longer than 1000 ms, the grouping benefit almost disappears. Our results suggest that the benefit of perceptual grouping occurs within the first 500 ms of stimuli presentation, and the grouping benefit on memory diminishes after 1000 ms presentation of stimuli. As we have mentioned before, this result suggests a spread of perceptual-level attention from grouped items to ungrouped items. In addition, although the pattern of variation in the grouping effect on VWM has not been reported before, there were findings that show a similar pattern of correlation between memory performance and duration of stimuli presentation. For example, Eng *et al*. found that the complexity of the memory items highly correlated with the memory performance with shorter display durations, and the correlation decreased with longer durations^69^. These findings suggest that memory performance is sensitive to the duration of stimuli presentation, and the results may have indications on the dynamics of the underlying mechanisms of encoding and processing.

Another interesting finding is that the effect of grouping seems to depend on grouping relevancy of the tested feature and presence of competition in the memory display. On the one hand, we found that grouping relevancy of the tested feature could moderate the the grouping effect on VWM, i.e., the grouping benefit significantly increases when the tested feature is relevant to grouping formation. On the other hand, the moderating effect of presence of competition is dependent on the grouping relevancy of the tested feature. If the tested feature is grouping relevant, whether or not to induce presence of competition in a memory display does not significantly change the grouping effect on VWM. However, if the tested feature is grouping irrelevant, employing a competition-present memory display is crucial for observing a strong and reliable grouping benefit. For example, suppose that there were several memory items with various shapes grouped by color similarity (e.g., red). If color was tested (memorizing red), its memory performance would be improved with or without presenting other colors in the memory display; but if shape was tested (memorizing the shape of red items), the performance would be significantly improved only if the grouped red items were presented simultaneously with the ungrouped items of various colors. The finding suggests that the benefit of perceptual grouping occurs at the encoding stage of working memory. It is possible that a grouping-relevant feature is detected and processed first in order to form perceptual grouping, and its storage could be inherent and independent of attention as the grouping process seems to occur at a pre-attentive stage^26–30^. The storage of such a feature does not involve competing for attention or memory resources with other features, because it may already be prioritized for processing and encoding in order for the perceptual group to be perceived. However, storage for a grouping-irrelevant feature should occur after the perceptual group is perceived. Since grouping-irrelevant features are perceptually equivalent to each other except that some belong to the grouped items and others belong to the ungrouped items, one may need to compete for resources with the others to obtain better storage. In this case, perceptual grouping may induce improved awareness and therefore increase the visual salience of the grouped items. Research shows that items with high visual salience can be better memorized^70–72^ by obtaining attentional priority in the encoding stage^62,71,73^. Therefore, with a competition-present display, those features belonged to the grouped items may gain more attention for processing and more resources for encoding through a process of ‘prior entry’, and thus are better stored. But with a competition-absent display, features all belong to the grouped items and are perceptually equivalent. Since there is no basis for prioritization, little grouping benefit can be observed. To summarize, our study suggests that the underlying mechanism of the grouping benefit is distinct with regard to grouping relevancy of the tested feature. If the tested feature is grouping relevant, the grouping benefit may occur at an early processing stage independent of attention; if the feature is grouping irrelevant, bottom-up attention may be driven by perceptual grouping and the grouping benefit may involve a process of ‘prior entry’ of the grouped items for more memory resources. The relationship between the grouping effect and grouping relevancy in combination with presence of competition has not been previously reported, and this finding suggests that the grouping effect on VWM relies critically on the characteristics of stimuli in relation to the memory display, and investigating the relationship between the two might provide us insights on the underlying mechanisms of the grouping benefit.

In addition, features like color and orientation yield better grouping effect than features like shape. This suggests that memory for certain features, regardless of their grouping relevancy, could benefit more from perceptual grouping. Conversely, it is also possible that features benefited more from perceptual grouping are those features that can induce perceptual grouping more effectively. For example, Quinlan & Wilton found that proximity and color similarity grouping have persuasive beneficial effect on performance for a discrimination task, while shape similarity grouping does not^23^. Indeed, although shape similarity has been frequently investigated in perceptual grouping discrimination tasks, the results were mixed: some research shows beneficial effects^21^, while others show weak or no effects^20,23^. This shows that features do differ in grouping effectiveness, and color may be a more salient feature for grouping than shape.

Our meta-analysis shows that employing a cue or a verbal suppression task does not seems to change the grouping effect. The former suggests that perceptual grouping seems to occur at a pre-attentive stage of visual processing, which is consistent with a number of studies^26–30^. Empirical research shows that the grouping effect on VWM exists in precue, postcue^18^, and no cue^5^ conditions, and the meta-analysis shows that the magnitude of the effect is not moderated by cue employment, i.e., whether explicitly directing attention to a grouped item does not vary the strength of the grouping effect. On the other hand, the non-significant effect found on verbal suppression is also consistent with several studies^67,74^. Indeed, visual tasks and verbal tasks seem to involve distinct processes independent of each other^74^. Since verbal suppression does not affect the memory performance, researchers may want to spare the trouble of employing such a task.

In addition to the above moderators, there may be other factors that potentially influence the grouping effect. For example, the duration of retention interval could affect the memory performance and might modulate the grouping effect, examining this factor may shed lights on how the grouping effect changes during the maintenance stage. However, since 37 out of 43 studies used a retention interval between 800 ms–1000 ms, the number of effect sizes for a longer retention is not sufficient to perform a reliable analysis. Moreover, research shows that perceptual grouping may only benefit VWM under certain circumstances. For example, Woodman *et al*. found that grouping affects the memory performance only if the number of to-be-remembered items exceeds the capacity limit of VWM (presumably, four)^18^. However, since more than half of our included studies used a set size equal or smaller than 4 and showed that VWM was improved by grouping, a subgroup analysis for set size is not necessary as there is already sufficient evidence demonstrating a grouping effect with small set sizes.

### Limitations

In our meta-analysis, we employed strict inclusion and exclusion criteria to select eligible studies, studies were coded systematically, effect sizes were computed with caution to obtain an overall effect size for grouping, and moderator analyses were performed for each potential influencing factors. However, there are still limitations in the present analyses. First, the number of included studies is not large. Although the major reason may be that there are indeed not that many studies being conducted on this specific topic, we speculate that there might also be some unpublished studies with no grouping effect, especially for those Gestalt principles that do not show a significant effect in our meta-analysis. Furthermore, our inclusion and exclusion criteria are stringent – the included studies must have a within-subject design, i.e., both the grouped and ungrouped items were tested on the same participants; perceptual grouping must be achieved by Gestalt principles – which might exclude a few potential studies. Second, in moderator analyses, there may be large error variance for a few subgroups that contained only two or three eligible studies with a small sample size. In particular, in the subgroup of *Common region*, only two studies were included with a total of 20 participants. This may result in a large sampling error and 95% CI, therefore even though the mean effect size is large (*d* = 0.97), it is not significant. Third, nineteen of the included studies did not explicitly report the mean or s.d. (17 only reported means and 2 reported neither), therefore we estimated these statistics based on figures of the results using a software *Get Data* (counting number of pixels between two target points). This may also cause manual estimation errors. Fourth, although we believe that the most prominent and important factors that influence the grouping effect have been captured, the search for moderators might not be exhaustive. Factors as discussed above may be evaluated given more eligible studies. Finally, the current study focuses only on healthy adults, thus we are uncertain whether the results can be generalized to other population like children, adolescents or clinical populations.

### Evidence from neuralphysiological studies

Besides the numerous behavioral studies that investigate the characteristics of visual working memory, neuralphysiological evidence also provides us insights into the neural substrates of visual memory processes. It is known that an event-related potential component, *contralateral delay*-*related activity* (CDA)^75^, reflects the encoding and maintenance of items in visual memory. The amplitude of CDA increases with the number of objects being held in the memory, and plateaus at a set size that predicts one’s VWM capacity limits^60,75^. Studies using CDA show that simple features such as color and orientation can be integrated into one representation in memory, therefore the CDA amplitudes are equivalent for a simple feature and a feature-feature conjunction^76,77^. However, during the retention interval, the CDA amplitudes are larger for complex objects than for simple objects, suggesting that the maintenance process is more challenging for complex objects^78^. In addition, Gao and his colleagues found that the CDA amplitude for four identical colors is comparable to the amplitude for one color, and both are lower than for four different colors^79,80^. This suggests that each perceptual group may count as a single ‘item’. Peterson *et al*. found a reduction in CDA amplitude when VWM is improved by grouping, suggesting that fewer neural resources are required to maintain grouped items, i.e. grouping induces more efficient processing^49^.

Functional magnetic resonance imaging (fMRI) data show that posterior parietal cortex is tightly correlated with the capacity of VWM, where the activity increases with set size^81^. Xu & Chun found that the inferior intra-parietal sulcus (IPS) maintains a fixed number of object representations at different spatial locations regardless of object complexity, and the superior IPS and the lateral occipital complex encode and maintain a variable subset of the attended objects, representing fewer objects as their complexity increases^82^. In a later study, they found that perceptual grouping reduces fMRI responses in inferior IPS even when grouping is task-irrelevant^37^. The authors suggest that the relative ease of selecting and representing grouped objects then allows more information to be encoded and stored in the superior IPS. However, the underlying mechanisms of grouping in VWM are not fully understood. Further neurophysiological research may contribute to our understanding of the mechanisms underlying the perceptual grouping benefits on VWM.

## Conclusions

Our empirical study found that grouping by illusory contour significantly improves VWM performance even when the tested feature is grouping irrelevant. The robust beneficial effect of grouping found in the meta-analysis suggests that perceptual grouping may help us to overcome the limitations of the extremely small capacity of VWM in daily tasks. More importantly, perceptual grouping seems to occur at a pre-attentive stage of visual processing, and the grouping benefit is developed within the first 500 ms presentation of stimuli, suggesting that the grouped items may be prioritized for processing and memorizing. In addition, our study suggests that the underlying mechanism of the grouping benefit may be different with regard to grouping relevancy of the to-be-stored feature. The grouping effect on VWM may be independent of attention for a grouping relevant feature, but may rely on attentional prioritization for a grouping irrelevant feature. This meta-analysis is the first research synthesis study that comprehensively summarize the effect of Gestalt grouping on visual working memory, and the results may serve to direct and focus the future research in related topics.
